# Accuracy of Estimation of Genomic Breeding Values in Pigs Using Low-Density Genotypes and Imputation

**DOI:** 10.1534/g3.114.010504

**Published:** 2014-02-13

**Authors:** Yvonne M. Badke, Ronald O. Bates, Catherine W. Ernst, Justin Fix, Juan P. Steibel

**Affiliations:** * Department of Animal Science, Michigan State University, East Lansing, Michigan 48824; ‡ Department of Fisheries & Wildlife, Michigan State University, East Lansing, Michigan 48824; † Smithfield Premium Genetics Group, Rose Hill, North Carolina 28458

**Keywords:** genomic selection, genotype imputation, swine, shared data resources, GenPred

## Abstract

Genomic selection has the potential to increase genetic progress. Genotype imputation of high-density single-nucleotide polymorphism (SNP) genotypes can improve the cost efficiency of genomic breeding value (GEBV) prediction for pig breeding. Consequently, the objectives of this work were to: (1) estimate accuracy of genomic evaluation and GEBV for three traits in a Yorkshire population and (2) quantify the loss of accuracy of genomic evaluation and GEBV when genotypes were imputed under two scenarios: a high-cost, high-accuracy scenario in which only selection candidates were imputed from a low-density platform and a low-cost, low-accuracy scenario in which all animals were imputed using a small reference panel of haplotypes. Phenotypes and genotypes obtained with the PorcineSNP60 BeadChip were available for 983 Yorkshire boars. Genotypes of selection candidates were masked and imputed using tagSNP in the GeneSeek Genomic Profiler (10K). Imputation was performed with BEAGLE using 128 or 1800 haplotypes as reference panels. GEBV were obtained through an animal-centric ridge regression model using de-regressed breeding values as response variables. Accuracy of genomic evaluation was estimated as the correlation between estimated breeding values and GEBV in a 10-fold cross validation design. Accuracy of genomic evaluation using observed genotypes was high for all traits (0.65−0.68). Using genotypes imputed from a large reference panel (accuracy: *R*^2^ = 0.95) for genomic evaluation did not significantly decrease accuracy, whereas a scenario with genotypes imputed from a small reference panel (*R*^2^ = 0.88) did show a significant decrease in accuracy. Genomic evaluation based on imputed genotypes in selection candidates can be implemented at a fraction of the cost of a genomic evaluation using observed genotypes and still yield virtually the same accuracy. On the other side, using a very small reference panel of haplotypes to impute training animals and candidates for selection results in lower accuracy of genomic evaluation.

Genetic improvement through breeding for lean growth, reproductive performance, meat quality, and health traits is an important tool in the pig-breeding industry to assure its continued competitiveness and success. Traditional estimated breeding values (EBVs) derived from pedigree information have resulted in continuous genetic improvement but have several limitations (Dekkers *et al.* 2010). Notably, some important phenotypes are difficult and expensive to observe, impairing estimation of accurate EBV.

The use of genomic breeding values (GEBVs), estimated using a large number of genetic markers across the genome, is expected to overcome a number of those limitations (Meuwissen *et al.* 2001; Dekkers *et al.* 2010) and allow for the selection of animals at a young age, thereby shortening generation intervals (Hayes *et al.* 2009a; Vanraden *et al.* 2009; Wiggans *et al.* 2011). Several papers have reported the progress and success of genomic selection in dairy cattle (Hayes *et al.* 2009a; VanRaden *et al.* 2009; Wiggans *et al.* 2011), and it is expected to be equally useful in pigs (Tribout *et al.* 2012). High-density genotypes in pigs can be obtained from the PorcineSNP60 BeadChip (Illumina, San Diego, CA) containing roughly 62K single-nucleotide polymorphisms (SNPs) (Ramos *et al.* 2009).

First implementations of genomic prediction in pigs included evaluations for total number of pigs born in a litter and percent stillborn (Cleveland *et al.* 2010). The results of this study indicated that GEBV in pigs can reach accuracies comparable with those observed in dairy cattle if the training population is large enough (Cleveland *et al.* 2010). In addition, several strategies to increase cost efficiency through the use of low-density genotypes have been explored, but the accuracy of GEBV was reasonable only for certain traits, likely due to differences in the genetic architecture of the traits (Cleveland *et al.* 2010). However, when genotypes were imputed with high accuracy, results for genomic evaluation were promising for several traits in a commercial pig population (Cleveland and Hickey 2013).

A question that was not investigated in those papers and that we want to answer in this study is how different imputation scenarios (of varying cost and accuracy) translate into accuracy of genomic predictions. The posed question is important because the relatively high genotyping cost per animal currently limits the widespread commercial use of high-density genotypes for genomic selection purposes in pigs. One strategy to improve the cost efficiency of genotyping schemes is the use of genotype imputation for a portion of the population. In the interest of cost efficiency, it is likely that selection candidates will not be genotyped using a high-density array such as the PorcineSNP60 but rather will be genotyped on a low-density array like the recently released GeneSeek Genomic Profiler for Porcine LD (GGP-Porcine: GeneSeek Inc., a Neogen Co., Lincoln, NE), a subset of the PorcineSNP60 containing roughly 10K SNP. We showed (Badke *et al.* 2013) that genotypes in pigs can be imputed from the GGP-Porcine to the PorcineSNP60 with accuracy of *R*^2^ = 0.88 using linkage disequilibrium (LD)-based imputation algorithms with a small reference panel of haplotypes (*N* = 128 haplotypes). We also showed that imputation accuracy can be further improved by adding animals to the reference panel (Badke *et al.* 2013), or in case of a pedigreed population, by exploiting Mendelian segregation and population-wide LD (Huang *et al.* 2012; Gualdrón Duarte *et al.* 2013). In this paper, we use genotypes imputed based on population wide LD, offering a strategy that can be applied universally in any population, for which a suitable reference panel can be assembled.

Our objective was to estimate the accuracy of genomic evaluation using observed or imputed genotypes. Moreover, we consider two contrasting imputation scenarios: (a) a higher-cost and high-accuracy scenario in which high-density genotypes from training animals and from a reference panel are used to impute genotypes in candidates for selection and (b) a low-cost and lower-accuracy scenario in which a small reference panel of high-density haplotypes is used to impute genotypes in training animals and candidates for selection.

## Materials and Methods

### Materials

#### Animals and genotypes:

Data used in this study were collected from 983 Yorkshire sires. A pedigree of 4092 individuals spanning 22 generations and including all 983 sires and their registered ancestors was available from the National Swine Registry (NSR). Of 983 genotyped sires, 575 had their sire genotyped as well, 341 had a grand sire, and 597 animals had at least one half sib among the 983 animals. The number of full sibs was much lower, and only 110 sires had a full sib genotyped. Details on these quantities can be found in Supporting Information, Figure S1. High-density genotypes for these animals were obtained from samples provided by the NSR. Genotyping was performed at a commercial laboratory (GeneSeek) using the Illumina PorcineSNP60 BeadChip. The same dataset was previously used to assess the effect of genotype imputation (Badke *et al.* 2013) and is publicly available at: https://www.msu.edu/~steibelj/JP_files/imputation.html. Animal protocols were approved by the Michigan State University All University Committee on Animal Use and Care (AUF# 03/09-046-00). Genotyping rate of at least 90% of both animals and SNP and a minor allele frequency (MAF) of at least 5% were required for genotypes to be included in the analysis, leaving a total of 41,248 markers in 983 animals. SNPs that were not assigned to an autosomal position in map build 10.2 were excluded from the analysis. It was our goal to estimate the GEBV of male offspring of a sire and since sires will not pass an X chromosome to their male offspring, these SNP do not contribute to the sons’ GEBV (VanRaden *et al.* 2009). In addition to genotypes for 983 Yorkshire sires, a set of 128 Yorkshire haplotypes was available as a reference panel for genotype imputation from a previous study (Badke *et al.* 2012). These haplotypes are also freely available at https://www.msu.edu/~steibelj/JP_files/LD_estimate.html, and details on the design and phasing can be found in Badke *et al.* (2012).

#### Phenotypes:

For every animal and their parents, EBVs and accuracies were obtained for three traits from NSR through their traditional genetic evaluation. These traits were: backfat thickness (BF), number of days to 250 lb (D250), and loin muscle area (LEA). Descriptive statistics of EBV and accuracies are presented in Table 1. All code and data used in this paper have been assembled into an R package, accessible at: http://tinyurl.com/MSURGEBV.

**Table 1:** Descriptive statistics of EBVs

	BF	D250	LEA
$E\bar{B}V$	−0.03	4.57	0.61
*^${\bar{r}}_{EBV}^{2}$^**^a^*	0.74	0.67	0.75
N *^b^*	965	936	938
*h*^2^	0.45	0.26	0.47

EBV, estimated breeding values; BF, backfat thickness; D250, number of days to 250 lb; LEA, loin muscle area.

a Average reliability of EBV.

b Number of animals with usable EBV.

### Methods

#### De-regression of breeding values:

De-regressed breeding values (dEBVs) were used as response variables throughout the analysis. We computed individual animal dEBVs and their weights (*w_i_*) with the parent average removed by following the procedure outlined by Garrick *et al.* (2009). We discarded records with a negative weight. The weight of an animal will only be below 0 if the unknown information content on this particular animal and its offspring is below 0, such that there is no individual information observed. This would be the case in a young animal, where all observed information came from ancestors and parents of this animal. To avoid double counting, these animals were eliminated from the analysis because they did not contribute individual information. After de-regression and filtering a total of 965, 936, and 938 animals remained for the traits BF, D250, and LEA, respectively.

#### Estimation of genomic relationship matrix:

The genomic relationship matrix was estimated from observed or imputed high density (~41 *K*) SNP genotypes. Genotypes were expressed as allelic dosage, which is the number of copies of the minor allele, such that genotypes were entered into a marker matrix **W** as a decimal number in the interval [0, 2]. We obtained matrix **Z** by subtracting twice the allelic frequency of the minor allele (*p_i_*), from columns of **W** (VanRaden 2008). The genomic relationship matrix was then calculated as:

$$\text{G}=\frac{\text{Z}{\text{Z}}^{\prime }}{2\sum_{i=1}^{M}p_{i}\left(1-p_{i}\right)}$$ (1)

where $2\sum_{i=1}^{M}p_{i}\left(1-p_{i}\right)$ is a normalizing constant (Wang *et al.* 2012) summing expected variances across markers scaling **G** toward the numerator relationship matrix (VanRaden 2008). The allele frequency *p_i_* was obtained using all available animals (N = 983). Average relatedness between animals was obtained from the row/column vectors of **G**. We quantified relatedness in this study as the average of the top 10 relationships observed within the **G** matrix (*re*/10). The choice of top 10 as opposed of another number is arbitrary but driven by the fact that each animal had a very limited number of close and distant relatives in the training set (Figure S1). Moreover, other studies have used this measure and proposed its inclusion in future work on genomic selection to promote comparability (Daetwyler *et al.* 2013).

#### Implementation of prediction model:

Using the genomic relationship matrix from equation (1), an animal-centric model for genomic evaluations can be written as:

$$\text{y}=1_{n}\mu +\text{a}+\text{e}$$ (2)

where **y** is the vector of dEBV, *μ* is the overall mean, **a** is the vector of *n* animal effects $\left(\text{a}∼N\left(0,\text{G}\sigma_{a}^{2}\right)\right)$, and **e** is a vector of random residuals $\left(\text{e}∼N\left(0,\text{R}\sigma_{e}^{2}\right)\right)$. The variance of the dEBV is $var\left(\text{y}\right)=\text{G}\sigma_{a}^{2}+\text{R}\sigma_{e}^{2}$, where **R** is a diagonal matrix with diagonal elements $R_{ii}=\frac{1}{w_{i}}$, the inverse of the weights of the dEBV (VanRaden *et al.* 2011). Equivalently, the information in **G** can also be included in the incidence matrix of the animal effects **a** as follows (Vazquez *et al.* 2010):

$$\text{y}=1_{n}\mu +\text{C}{\text{a}}^{*}+\text{e}$$ (3)

where **C** is the Cholesky decomposition of **G**, such that **G** = **CC**′, *μ* is the overall mean, a* is the vector of animal effects with ${\text{a}}^{*}∼N\left(0,\text{I}\sigma_{a^{*}}^{2}\right)$ noticing that **a** = Ca*, and **e** is a vector of residual effects $\text{e}∼N\left(0,\text{R}\sigma_{e}^{2}\right)$ such that $var\left(\text{y}\right)=\text{C}{\text{C}}^{\prime }\sigma_{a^{*}}^{2}+\text{R}\sigma_{e}^{2}=\text{G}\sigma_{a^{*}}^{2}+\text{R}\sigma_{e}^{2}$. The variance terms for models (2) and (3) are equal, such that the two models are in fact equivalent if variance components are assumed known. Likewise, when estimating the parameters under these two models, we found virtually identical results, but model (3) was computationally more efficient resulting in a twofold reduction in compute time (results not shown). The BLR package (Pérez *et al.* 2010) in R (R Development Core Team 2011) was used to fit the mixed model equations. Model parameters $\sigma_{e}^{2}$ and $\sigma_{a*}^{2}$ were sampled from their corresponding full conditional distribution using a Gibbs sampler. Prior distributions were elicited based on equations presented by Pérez *et al.* (2010). The prior distribution of $\sigma_{e}^{2}$ and $\sigma_{a*}^{2}$ were an inverse *χ*^2^ distribution with degrees of freedom *df* and scale *S*. To ensure proper priors with finite expectations, we set *df* = 3. The scale parameters were obtained as a function of the *df* and assuming values of the genetic variance (*V_a_*) and error variance (*V_e_*) (Pérez *et al.* 2010):

$$\sigma_{e}^{2}∼\chi^{-2}\left(df_{e}=3,S_{e}=V_{e}\left(df_{e}+2\right)\right)$$

$$\sigma_{a*}^{2}∼\chi^{-2}\left(df_{a}=3,S_{a}=\frac{V_{a}\left(df_{a}+2\right)}{\bar{A_{ii}}}\right)$$

where $\bar{A_{ii}}$, is the average inbreeding coefficient, set equal to 1 in this case, assuming no inbreeding. Heritability was assumed to be *h*^2^ = 0.5, such that after the value for *V_e_* was arbitrarily set to 0.4, *V_a_* was estimated $V_{a}=\frac{V_{e}h^{2}}{1-h^{2}}$. The Gibbs sampler implemented in BLR (Pérez *et al.* 2010) was used to obtain a total of 100,000 samples, 10,000 of which were discarded as burn-in. The reported estimates of $\sigma_{e}^{2}$, $\sigma_{a*}^{2}$, animal effects (a*), and GEBV $\left(\hat{\text{y}}\right)$ were based on the posterior means of the remaining 90,000 iterations. We assessed convergence of the Markov chain Monte Carlo method as well as sensitivity to priors to ensure robustness of estimates to priors (results not shown).

### Genomic prediction under cross-validation

Accuracy of genomic evaluation was estimated in a 10-fold cross-validation design. Approximately 10% of the animals were randomly assigned to a validation panel (*V*) in which predictions would be made, whereas the remaining 90% were used as the training panel (*T*) to estimate the parameters necessary for prediction. A total of 10 separate datasets were created such that each animal would be used for validation once. Across cross-validation datasets we fit model (3) to the training animals; we refer to that subset by adding a subindex *T*:

$${\text{y}}_{T}=1_{n_{T}}\mu +{\text{C}}_{T}{\text{a}}_{T}^{*}+{\text{e}}_{T}$$ (4)

to estimate the BLUP of ${\hat{\text{a}}}_{T}^{*}$ (VanRaden *et al.* 2011):

$${\hat{\text{a}}}_{T}^{*}=C_{T}^{\prime }{\left({\text{G}}_{T}+{\text{R}}_{T}\frac{\sigma_{e}^{2}}{\sigma_{a}^{2}}\right)}^{-1}\left({\text{y}}_{T}-1_{n_{T}}\hat{\mu }\right)$$ (5)

where the matrices **G** and **C** are partitioned into block structure such that

$$\left[\begin{array}{cc} {\text{G}}_{T} & {\text{G}}_{TV}^{\prime }\\ {\text{G}}_{TV} & {\text{G}}_{V} \end{array}\right]=\left[\begin{array}{cc} {\text{C}}_{T} & 0\\ {\text{C}}_{TV} & {\text{C}}_{V} \end{array}\right]\left[\begin{array}{cc} {\text{C}}_{T}^{\prime } & {\text{C}}_{TV}^{\prime }\\ 0 & {\text{C}}_{V}^{\prime } \end{array}\right]=\left[\begin{array}{cc} {\text{C}}_{T}{\text{C}}_{T}^{\prime } & {\text{C}}_{T}{\text{C}}_{TV}^{\prime }\\ {\text{C}}_{TV}{\text{C}}_{T}^{\prime } & {\text{C}}_{TV}{\text{C}}_{TV}^{\prime }+{\text{C}}_{V}{\text{C}}_{V}^{\prime } \end{array}\right]$$ (6)

The relation between the BLUP for **a** based on model (2) and ${\hat{\text{a}}}^{*}$ based on model (3) can be expressed as:

$$\left[\begin{array}{c} {\text{a}}_{T}\\ {\text{a}}_{V} \end{array}\right]=\left[\begin{array}{cc} {\text{C}}_{T} & 0\\ {\text{C}}_{TV} & {\text{C}}_{V} \end{array}\right]\left[\begin{array}{c} {\text{a}}_{T}^{*}\\ {\text{a}}_{V}^{*} \end{array}\right]$$ (7)

The GEBVs of training animals in model (2) were computed as:

$${\hat{\text{a}}}_{T}={\text{C}}_{T}{\hat{\text{a}}}_{T}^{*}={\text{C}}_{T}{\text{C}}_{T}^{\prime }{\left({\text{G}}_{T}+{\text{R}}_{T}\frac{\sigma_{e}^{2}}{\sigma_{a}^{2}}\right)}^{-1}\left({\text{y}}_{T}-1_{n_{T}}\hat{\mu }\right)={\text{G}}_{T}{\left({\text{G}}_{T}+{\text{R}}_{T}\frac{\sigma_{e}^{2}}{\sigma_{a}^{2}}\right)}^{-1}\left({\text{y}}_{T}-1_{n_{T}}\hat{\mu }\right)$$ (8)

Subsequently, the GEBVs of the validation animals ${\hat{\text{a}}}_{V}$ were estimated from ${\hat{\text{a}}}_{T}$ using the following equation:

$${\hat{\text{a}}}_{V}={\text{G}}_{TV}{\text{G}}_{T}^{-1}{\hat{\text{a}}}_{T}={\text{C}}_{TV}{\text{C}}_{T}^{\prime }{\left({\text{G}}_{T}+{\text{R}}_{T}\frac{\sigma_{e}^{2}}{\sigma_{a}^{2}}\right)}^{-1}\left({\text{y}}_{T}-1_{n_{T}}\hat{\mu }\right)$$ (8)

where $\sigma_{e}^{2}$, $\sigma_{a}^{2}$, and $\hat{\mu }$ are estimated using model (4), which is equivalent to applying model (3) to the training animals.

#### Estimation of accuracy:

Accuracy of genomic evaluation is the correlation between the estimated GEBV and the unknown true breeding values (TBVs) (Hayes *et al.* 2009a). However, the TBVs are unknown. Consequently, the accuracy of genomic evaluation has to be approximated using the available information. Hayes *et al.* (2009a) proposed to express the correlation between GEBV and TBV as a function of the correlation between GEBV and EBV:

$$r_{\left(GEBV,TBV\right)}=\frac{cor\left(GEBV,EBV\right)}{cor\left(EBV,TBV\right)}=\frac{cor\left(GEBV,EBV\right)}{\sqrt{r_{EBV}^{2}}}$$ (9)

where $r_{EBV}^{2}$ is the estimated reliability of the EBV. VanRaden *et al.* (2009) replaced $r_{EBV}^{2}$ with the arithmetic mean of the reliability of the EBV. Daetwyler *et al.* (2013) proposed to report a simple Pearson correlation coefficient between GEBV and EBV to allow for comparability of results across studies. We estimate accuracy of genomic evaluation as the Pearson correlation coefficient between GEBV and EBV (*r*_(_*_GEBV_*_,_ *_EBV_*_)_) and the Pearson correlation coefficient adjusted for the average accuracy of the EBV to facilitate such comparison $\left(\frac{r_{\left(GEBV,EBV\right)}}{{\bar{r}}_{EBV}}\right)$.

Accuracies of individual GEBV were obtained analogous to the accuracy of EBV in an animal model (Goddard *et al.* 2011) through inversion of the mixed model equations (Mrode 2005; VanRaden 2008; VanRaden *et al.* 2009; Strandén and Garrick 2009; Clark *et al.* 2012). The accuracy of ${\hat{\text{a}}}_{V}$ of the model (2) can be expressed as (Mrode 2005; Strandén and Garrick 2009; Clark *et al.* 2012):

$$r_{{\hat{\text{a}}}_{V}}=\sqrt{\frac{{\left\{{\text{G}}_{TV}{\left({\text{G}}_{T}+{\text{R}}_{T}\frac{\sigma_{e}^{2}}{\sigma_{a}^{2}}\right)}^{-1}G_{TV}^{\prime }\right\}}_{ii}}{{\left\{{\text{G}}_{V}\right\}}_{ii}}}$$ (10)

This equation and its derivation can be found in Strandén and Garrick (2009) and VanRaden (2008) and was used to estimate the accuracy of individual GEBV for validation animals.

#### Genotype imputation:

LD-based genotype imputation was performed with BEAGLE version 3.3.1 (Browning and Browning 2009). We used the standard settings for BEAGLE: 10 iterations of the phasing algorithm, drawing four samples per iteration. Previous results from our group (Badke *et al.* 2013) and other studies (Hayes *et al.* 2012) showed negligible improvement in imputation accuracy as a result of an increase in iterations or samples per iteration. Imputation of 10K SNP chip [6890 SNP after filtering for minor allele frequency (MAF) and missing rate] were used as tagSNP to impute 60K SNP (41,248 after filtering).

We implemented two separate imputation experiments that differ in the size of the high-density reference panel used for imputation: (1) a reference panel of 128 Yorkshire haplotypes or (2) a reference panel combining the 128 Yorkshire haplotypes with the haplotypes of all animals that are part of the training panel (~1700 additional haplotypes) in the respective cross-validation dataset. To assess the effect of genotype imputation on genomic prediction we considered the following four scenarios: (1) the reference scenario in which genomic evaluation was based on observed genotypes in training and validation animals, (2) genomic evaluation based on observed genotypes in the training animals and genotypes imputed from a large reference panel (~1800 haplotypes) in the validation animals, (3) genomic evaluation based on observed genotypes in the training animals and genotypes imputed from a small reference panel (128 haplotypes) in the validation animals, and (4) genomic evaluation based on imputed genotypes in training and validation animals using a small (128 haplotypes) but representative reference panel for imputation. All genotype imputation and subsequent estimation of imputation accuracy was implemented using the R package impute.R (Badke *et al.* 2013). To compare average accuracy of genomic evaluation across these four scenarios, we fitted a linear model with the average accuracy of genomic evaluation as response variable and the genotype imputation scenario as independent variable, adding the effect of the random cross-validation dataset in which accuracy of genomic evaluation was estimated as a random blocking factor.

## Results

### Accuracy of genomic evaluation and GEBV using observed genotypes

When genotypes were observed in both training and prediction animals, the accuracy of genomic evaluation, measured as the weighted mean of the Pearson correlation coefficient between EBV and predicted GEBV across 10 cross-validation datasets, was 0.68, 0.66, and 0.65 for BF, D250, and LEA, respectively (Table 2). When the measure of accuracy was adjusted for the average reliability of the EBV of the training animals, the observed accuracy of genomic evaluation was 0.80, 0.82, and 0.76 for BF, D250, and LEA, respectively (Table 2).

**Table 2:** Estimates of accuracy for genomic evaluation and individual GEBV across imputation scenarios

Trait	Scenario*^a^*	Imputation Accuracy*^b^*	*r_EBV_*_,_ *_GEBV_**^c^*	*r*_EBV_*^d^*	$\frac{r_{EBV,GEBV}}{{\bar{r}}_{EBV}}$	${\bar{r}}_{GEBV}$	*HPD**^e^*
BF	1	(1, 1)	0.6810^1^	0.8510	0.7998	0.6852	[0.5395, 0.8211]
	2	(1, 0.95)	0.6795^1^		0.7981	0.6861	[0.5467, 0.8164]
	3	(0.88, 0.88)	0.6598^2^		0.7749	0.7014	[0.5727, 0.8267]
	4*^f^*	(1,1)	0.7210		0.8405	0.8560	[0.8174, 0.8768]
D250	1	(1, 1)	0.6603^1^	0.8020	0.8229	0.6575	[0.5073, 0.7948]
	2	(1, 0.95)	0.6555^1,^^2^		0.8170	0.6585	[0.5187, 0.7962]
	3	(0.88, 0.88)	0.6463^2^		0.8054	0.6750	[0.5345, 0.7985]
	4*^f^*	(1,1)	0.5354		0.6550	0.8438	[0.8048, 0.8704]
LEA	1	(1, 1)	0.6516^1^	0.8529	0.7639	0.6859	[0.5386, 0.8325]
	2	(1, 0.95)	0.6491^1^		0.7610	0.6868	[0.5377, 0.8214]
	3	(0.88, 0.88)	0.6364^2^		0.7461	0.7040	[0.5667, 0.8330]
	4*^f^*	(1,1)	0.7165		0.8201	0.8549	[0.8223, 0.8787]

GEBV, genomic breeding value; EBV, estimated breeding values; HPD, highest posterior density; BF, backfat thickness; D250, number of days to 250 lb; LEA, loin muscle area.

a Scenarios 1: all observed genotypes, 2: genotypes in prediction animals imputed with large reference haplotype panel (~1800), 3: genotypes in prediction animals imputed with small haplotype reference panel (128), and 4: validation animals with at least one close relative in the reference panel.

b Accuracy of genotype imputation *R*^2^ for training and validation animals: $\left(R_{T}^{2},R_{V}^{2}\right)$.

c Tukey honest significant difference post-hoc comparison of accuracy of genomic evaluation across imputation scenarios.

d Average accuracy of EBV in the validation panel.

e 95% HPD interval of GEBV accuracy across validation animals.

f Scenario with young animals in the validation panel that almost all have at least one close relative in the training panel.

^1,2^Means with different superscript differ significantly according to Tukey post-hoc tests with *α* = 0.05.

We observed a significant difference between the estimates of accuracy of genomic evaluation across 10 randomly assigned cross-validation datasets for three traits (Table 3). That variation across cross-validation datasets was partially explained by a significant effect of the average EBV accuracy of validation animals on accuracy of genomic evaluation (Table 3) in three traits and a significant effect of top 10 relatedness on accuracy of genomic evaluation in D250. In general, D250 had slightly lower average EBV accuracy due to an increased frequency of EBV with intermediate accuracy (*r_EBV_* close to 0.6, Figure S2). As expected, this resulted in slightly lower correlation of EBV and GEBV because the ‘true value’ (EBV) is subject to more uncertainty. Another source of difference of accuracy of genomic evaluation across cross-validation datasets could be the population structure. This would be revealed through differences in estimated variance components. We did not expected differences in variance components estimated from randomly assigned validation datasets. We confirmed this assumption by studying the distribution of estimated heritability $\left(\frac{\sigma_{a}^{2}}{\sigma_{a}^{2}+\sigma_{e}^{2}}\right)$ and included the obtained results in Figure S3. We observed that the posterior distributions of heritabilities did not change across folds. Conversely, in the presence of population structure, the relationships of animals of different cross validation datasets will change (depending on who else is in the training set), and we expect that to affect the estimate of heritability.

**Table 3:** Significance of variables affecting accuracy of genomic evaluation

dataset*^a^*			*rel10**^b^*		*^${\bar{r}}_{EBV}$^**^c^*	
trait	*F**^d^*	*p*	*F*^e^**	*p*	*F^e^*	*p*
BF	258	< 0.001	2.83	0.1013	11.73	0.0016**
D250	229	< 0.001	5.18	0.0291*	7.238	0.0109*
LEA	311	< 0.001	2.06	0.1605	3.430	0.0725

EBV, estimated breeding values; BF, backfat thickness; D250, number of days to 250 lb; LEA, loin muscle area.

a Accuracy of genomic evaluation was estimated for a total of 10 randomly assigned datasets of the cross-validation, such that we could assess whether accuracy of genomic evaluation was significantly different across these 10 datasets.

b Accuracy of genomic evaluation by average of the top 10 genomic relationship estimates of animals in the validation set.

c Accuracy of genomic evaluation by average accuracy of EBV of validation animals by cross-validation dataset.

d *df* = *c*(9, 27).

e *df* = *c*(1, 35).

* *P* < 0.05, **P < 0.01.

The average accuracy of the genomic evaluation and the assessment of the accuracy of individual GEBV using equation 10 is equally important in a practical implementation of genomic selection. Average accuracy of individual GEBV was 0.69, 0.66, and 0.69 for BF, D250, and LEA, respectively with a 95% highest posterior density interval ranging from roughly 0.51 to 0.80 across all traits (Table 2).

**Figure 1:**
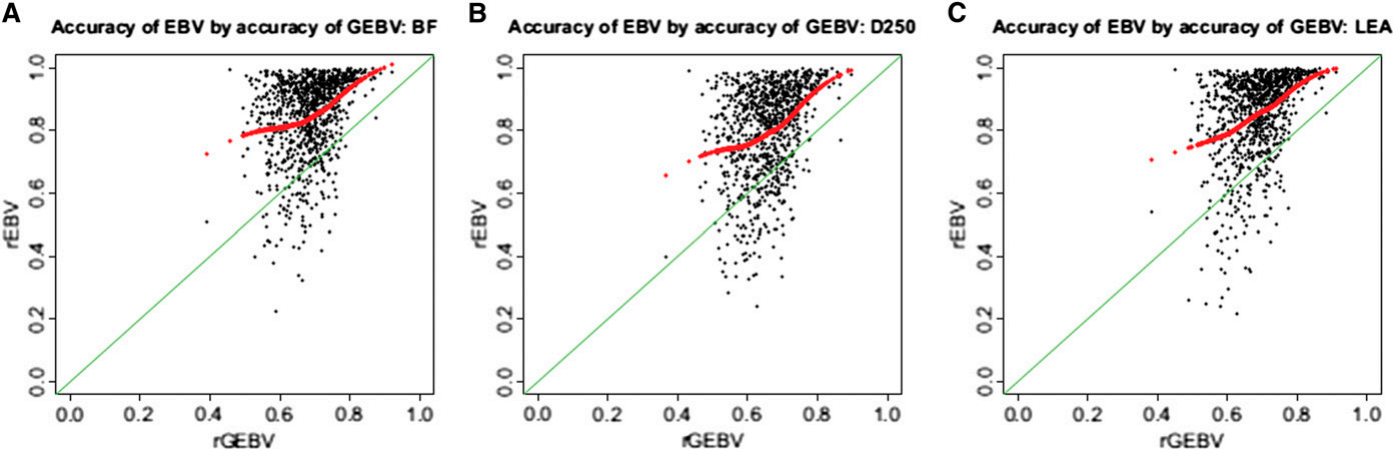
Accuracy of GEBV by observed accuracy of EBV for (A) BF, (B) D250, and (C) LEA *r_GEBV_* in relation to the animals *r_EBV_*, with the 1-1 line of the regression (green line) and a loess smoother (red line), which is a local weighted mean of the *r_GEBV_*. GEBV, genomic breeding value; EBV, estimated breeding value; BF, backfat thickness; D250, number of days to 250 lb; LEA, loin muscle area.

**Figure 2:**
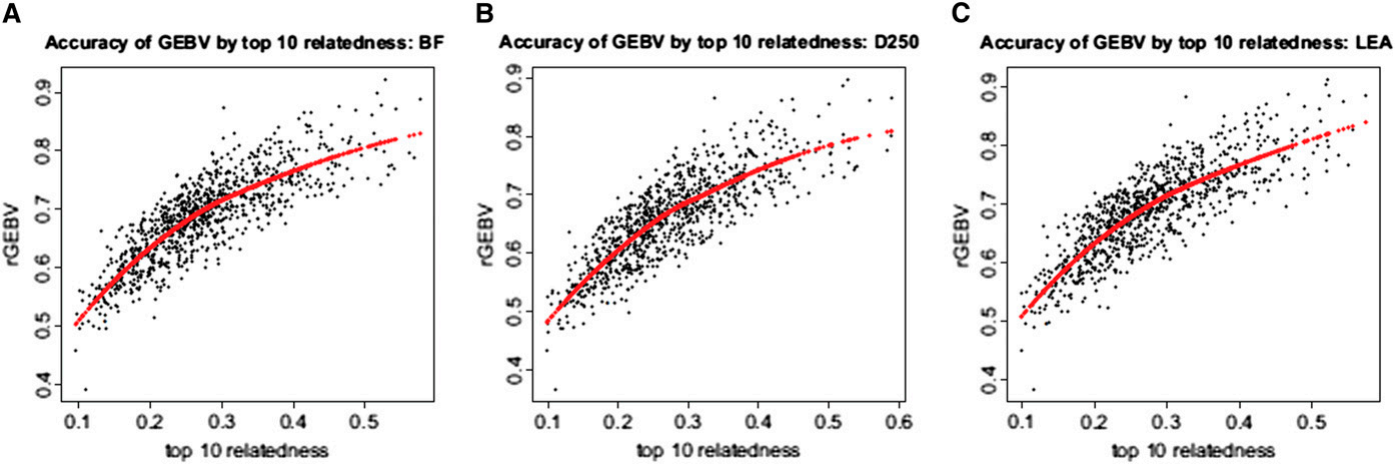
Accuracy of GEBV by average top 10 relatedness between the individual and training panel for (A) BF, (B) D250, and (C) LEA *r_GEBV_* in relation to the animals *rel*10, a loess smoother (red line), which is a local weighted mean of the *r_GEBV_*. GEBV, genomic breeding value; BF, backfat thickness; D250, number of days to 250 lb; LEA, loin muscle area.

As can be seen in Figure 1, the accuracy of GEBV (*r_GEBV_*) and accuracy of EBV (*r_EBV_*) are not linearly related. The accuracy of EBV was higher than the estimated accuracy of GEBV for most animals in three traits, especially when *r_EBV_* > 0.8. For a few animals with *r_EBV_* between 0.4 and 0.8, the accuracy of GEBV was higher than their respective EBV accuracy. Hypothetically, individual differences in *r_GEBV_* can be explained by the presence or absence of relatives of the predicted animal in the training set (Clark *et al.* 2012; Pérez-Cabal *et al.* 2012). We investigated this assertion in two ways: (1) by computing average *r_GEBV_* for animals with different number of relatives in training panel and (2) by regressing *r_GEBV_* on the average top 10 relatedness in the genomic relationship matrix. Following Pérez-Cabal *et al.* (2012), we defined close relatives as sires and full sibs and distant relatives as maternal grand sires and half sibs. We found that increasing the number of close relatives from one to four in the training panel increased average *r_GEBV_* by about 0.1 decimal points (Figure 2) across the three traits in this study (from an average of = 0.63 to = 0.73 regardless of the trait considered). The presence of distant relatives in the training set also resulted in an increase of *r_GEBV_* of similar magnitude when comparing individuals without any distant relative to individuals with at least five distant relatives in the training set (Figure 2). A similar relationship was observed when comparing *r_GEBV_* with the average relationship to the 10 most-related individuals in the training set. We observed an almost linear increase in *r_GEBV_* as top 10 relatedness increased (Figure 2), which was statistically significant (*P* < 0.01). To further investigate the effect of relatedness between training and validation animals, we selected the youngest 87 animals (approximately 10% of the population) that included 82 animals with at least a sire or a grand-sire in the training panel. We repeated genomic evaluation with this validation panel and estimated the accuracy of GEBV. As expected, average accuracy of GEBV for this validation panel was higher than the average observed across the cross-validation datasets with 0.72 for BF and LEA, and 0.54 for D250. However, when looking at the range of accuracies observed for all 10 cross-validation datasets these values do not exceed the maximum accuracy observed. One interesting finding was that estimates of individual accuracy, or accuracy of GEBV predicted through the genomic relationship matrix, were much larger than the observed accuracy of genomic evaluation in all three traits (Table 2). Goddard *et al.* (2011) proposed to use this measure of accuracy of individual GEBV when using them for selection but also to screen for animals whose GEBV could be expected to be highly accurate. Our results show that while it is true that individuals with close relatives in the training panel will have on average more accurate GEBV, the individual accuracies obtained from the **G** matrix would be overestimated.

### Effect of genotype imputation on accuracy of genomic evaluation and GEBV

Accuracy of imputation (*R*^2^) for each animal was measured as the squared correlation between the observed and imputed allelic dosage across all SNP (Badke *et al.* 2013). Average accuracy of imputation was *R*^2^ = 0.88 for the scenario using a small (128) haplotype reference panel, and it increased to *R*^2^ = 0.95, when a larger reference panel (~ 1800 haplotypes) was used. In our previous study (Badke *et al.* 2013), we found that increasing the size of the reference panel led to an improved imputation, especially of SNP that appear difficult to impute, such as SNP with low (<0.1) MAF and those located in the chromosomal extremes. These results were repeated in this study (Figure S4). For BF we found that the average accuracy of genomic evaluation under scenario 2 (*r_GEBV_*_,_ *_EBV_* = 0.68), where genotypes in the validation animals were imputed with high accuracy (*R*^2^ = 0.95), was not significantly different from the accuracy (*r_GEBV_*_,_ *_EBV_* = 0.68) estimated in the reference scenario, where all genotypes were observed. However, average accuracy of genomic evaluation was significantly lower (*r_GEBV_*_,_ *_EBV_* = 0.66), when genotypes were imputed in both training and validation with lower accuracy (*R*^2^ = 0.88 using a small reference panel of haplotypes (scenario 3). For D250, there was no significant difference in accuracy of genomic evaluation between the reference scenario (*r_GEBV_*_,_ *_EBV_* = 0.66) and the scenario where genotypes were imputed in the validation animals (Table 2). However, when genotypes were imputed in both training and validation (scenario 3), the accuracy of genomic selection was significantly lower (*r_GEBV_*_,_ *_EBV_* = 0.65). For LEA there was also no difference in accuracy of genomic evaluation between the reference scenario (*r_GEBV_*_,_ *_EBV_* = 0.65) and scenario 2 (*r_GEBV_*_,_ *_EBV_* = 0.65). There was a significant decrease in accuracy of genomic evaluation when genotypes were imputed with lower accuracy (*R*^2^ = 0.88) in scenario 3 (*r_GEBV_*_,_ *_EBV_* = 0.63). To assess the effect of genotype imputation on the results of a genomic evaluation, we compared the top 5% sires (*n* = 46), ranked by their estimated GEBV across imputation scenarios. Again, scenario 1 was used as a reference scenario to compare how many of the top 5% ranked animals were also top ranked under the imputation scenarios. The proportion of top 5% ranked sires that were conserved when genotypes were imputed in validation animals with high accuracy (scenario 2) was 0.96 for BF and 0.98 for D250 and LEA. When genotypes were imputed with low accuracy in training and validation, the proportion of top 5% sires conserved in comparison with the reference design showed a small decrease compared with the design with only validation animals imputed for BF (0.88) and for D250 (0.89), and a more substantial decrease for LEA (0.81). Accuracy of individual GEBV is estimated using the genomic relatedness between training and validation animals. Using genotypes imputed with high accuracy (*R*^2^ = 0.95) the estimated *r_GEBV_* remained constant in all traits, compared with estimates obtained from observed genotypes. Accuracy of imputation was correlated with *r_GEBV_* (Figure S5). However, this does not imply that high imputation accuracy caused an increase in *r_GEBV_*. Another possibility is that genotypes from animals with relatives in the reference panel will be imputed with high accuracy and their GEBV will also be predicted more accurately. We believe that this was the case for our population because the correlation between GEBV and EBV did not differ significantly when imputation was used (Table 2, compare scenario 1 and 2). Moreover, when genotypes were imputed with less accuracy (*R*^2^ = 0.88), the observed accuracy of GEBV was increased even with respect to the reference scenario (Table 2, compare scenario 3 to 1 and 2). This result is counterintuitive, and we investigated the reason for this increase. Examining the estimation procedure for *r_GEBV_* we found that the increase was due to smaller estimates of the diagonal elements of the genomic relationship matrix between the validation elements (**G***_V_*) in the scenario with all imputed genotypes. This is the result of all imputed animals conditional on a small reference panel looking genetically more similar than they really are (because they are all imputed toward the haplotype frequencies in the small panel). Those diagonal elements of **G** were used to scale values of *r_GEBV_* (equation 10), and smaller values in the denominator resulted in the larger estimates of *r_GEBV_* we saw for animals in scenario 3. Comparing unscaled values of *r_GEBV_* individual accuracy was higher in the reference scenario for all animals.

## Discussion

### Accuracy of genomic evaluation and GEBV using observed genotypes

The size of the training population used to train the prediction equation in this study was small compared with previous genomic evaluations published in swine (Cleveland *et al.* 2010, 2012), and especially compared with studies applying genomic evaluation in European (Dassonneville *et al.* 2011) or US dairy cattle (Weigel *et al.* 2010; Wiggans *et al.* 2012). Observed accuracy of genomic evaluation in this study was in good agreement with previously published results for genomic evaluation in pigs, assessing five unspecified commercial traits with comparable heritability (Cleveland *et al.* 2012) and earlier results for two reproductive traits (Cleveland *et al.* 2010). Accuracy of genomic evaluation was high across three traits (BF: *r_GEBV_* = 0.6810; D250: *r_GEBV_* = 0.6603; LEA: *r_GEBV_* = 0.6516). In addition, we report accuracy adjusted for the fact that the Pearson correlation between EBV and GEBV will underestimate the true quantity of interest (Luan *et al.* 2009). Assessing the variation in accuracy of genomic evaluation across datasets of the cross-validation, we found that the ${\bar{r}}_{EBV}$ of the validation animals and their relatedness to the training animals were significantly associated to the average accuracy of genomic evaluation. Higher accuracy of genomic evaluation of prediction animals with close relatives in the training population (Habier *et al.* 2010; Clark *et al.* 2012) and within closely related populations, with relatively small effective population size, has been previously reported (Daetwyler *et al.* 2013). Accuracy of genomic evaluation in this study was high despite the limited number of animals available for training and the inclusion of animals with relatively low EBV accuracy. Furthermore, we obtained accurate genomic predictions using an equivalent model fitting the genomic relationship matrix instead of a marker based matrix (Hayes *et al.* 2009b), thereby greatly reducing the computational load. We expect that accuracy of genomic evaluation in this population and other US swine populations with comparable population structure and LD (Badke *et al.* 2012), will be feasible for commercial implementation and could be further increased through the inclusion of additional training animals with highly accurate EBV.

Besides assessing the accuracy of genomic evaluation, we also reported accuracies for individual GEBV. The accuracy of GEBV is important because it can influence selection decisions. Moreover, as proposed by Goddard *et al.* (2011), *r_GEBV_* can also be approximated prior to the implementation of genomic evaluation and used to inform the design of genomic selection in a population. The main difference between *r_GEBV_* and *r*_(_*_GEBV_*_,_ *_EBV_*_)_ is that *r*_(_*_GEBV_*_,_ *_EBV_*_)_ is indicative of the average accuracy of GEBV in a population, whereas *r_GEBV_* gives a measure of accuracy of each individually estimated GEBV. As expected, we observed that accuracy of GEBV increased with increased relatedness between the animal and the training panel. An interesting finding was that under a low accuracy imputation scenario, *r_GEBV_* was overestimated compared with *r*_(_*_GEBV_*_,_ *_EBV_*_)_. We traced this back to the diagonal elements of the genomic relationship matrix and attributed it to an artifact of the imputation using a small reference panel.

Several previous studies in other populations and simulation experiments also showed the importance of relatedness for the prediction of accurate GEBV (Habier *et al.* 2010; Clark *et al.* 2012), especially when the training population was small (Wientjes *et al.* 2013) as was the case in our study. In addition, we observed that accuracy of GEBV was higher than accuracy of EBV for only a few animals that had mostly low accuracy of EBV. This finding is further supported by previous reports that implementation of genomic evaluation would be most beneficial for young animals with little information on their own and subsequently low accuracy of traditional EBV (VanRaden 2008).

### Effect of genotype imputation on accuracy of genomic evaluation and GEBV

Genotype imputation is an efficient tool to decrease the cost of obtaining high-density genotypes for selection candidates. One of the goals of this study was to quantify the loss on accuracy of genomic evaluation if GEBV were estimated from imputed rather than observed genotypes in selection candidates. Comparing accuracy of genomic evaluation across three scenarios of genotype imputation we found that for three traits there was no significant loss of accuracy of genomic prediction if genotypes in validation animals were with high accuracy (*R*^2^ = 0.95) instead of observed. However, accuracy of genomic evaluation decreased in comparison with the reference scenario when genotypes were imputed with lower overall accuracy (*R*^2^ = 0.88). When low-accuracy imputation was applied in training and prediction animals we observed a decrease in accuracy of genomic evaluation. Previously published results support that although it is not feasibly to implement genomic prediction based on low-density genotypes (Habier *et al.* 2009; Cleveland *et al.* 2010) the accuracy of genomic evaluation is still feasible for practical implementation when genotypes in selection candidates are accurately imputed to high density (Weigel *et al.* 2010; Cleveland and Hickey 2013). In addition, several studies also support that an increase in imputation accuracy will generate genomic evaluations with nearly identical or even higher accuracy compared with that obtained from observed genotypes (Dassonneville *et al.* 2011; Wiggans *et al.* 2012; Cleveland and Hickey 2013) because the cost efficiency of low-density genotypes allows a much larger proportion of the population to be included in the genomic evaluation procedure (Wiggans *et al.* 2012). In conclusion, an implementation of genomic selection based on observed genotypes for training of the prediction equation and GEBV predictions obtained from genotypes imputed with high accuracy appears to be a promising approach to provide the swine breeding industry with a cost-efficient procedure to obtain GEBV for animals at a young age. A recent study assessing the accuracy of genomic evaluation using high-density genotypes and various imputation schemes in a commercial pig population further supports these findings (Cleveland and Hickey 2013).

We found that accuracy of individual GEBV was a linear function of the relatedness between a validation animal and the respective training set. As has been previously shown in the literature, animals that are highly related to the training population will have higher *r_GEBV_* (Habier *et al.* 2010; Clark *et al.* 2012). As shown in the last scenario, however, when all selection candidates had at least one close relative in the training population, *r_GEBV_* overestimates the accuracy observed for the genomic evaluation (*r*_(_*_EBV_*_,_ *_GEBV_*_)_). Although this measure certainly has value to rank animals according to how trustworthy estimated GEBV are, it is likely overestimated for candidates with close relatives.

The other case in which we observed overestimated individual accuracy of GEBV (*r_GEBV_*) pertains to the last of the imputation scenarios where genotypes were imputed in training and prediction animals. Specifically, when genotypes were imputed in training and prediction animals with lower accuracy, the average *r_GEBV_* was larger than the accuracy of genomic evaluation, which we found was an artifact of lower estimates of the diagonal elements of the **G** matrix. This was caused by a decrease in the variance of the allelic dosage of imputed genotypes due to the relatively small number of reference haplotypes available. When the variance of imputed allelic dosages was decreased, the deviation from the expected value estimated from MAF (2**p**) also decreased, causing overall smaller estimates of **Z** and the resulting diagonal elements of the **G** matrix. This increase in the homogeneity of allelic dosages in the imputed genotypes causes the observed inflation in accuracy of estimated GEBV, such that in any case when GEBV are obtained from imputed genotypes the estimated accuracy of GEBV should be used with caution. The average GEBV accuracy notably exceeded the expected accuracy of genomic evaluation in that scenario.

In conclusion, we found that results for the accuracy of GEBV further support the notion that genomic evaluation using high-density genotypes imputed with high accuracy for selection candidates is a feasible method to implement a cost-efficient design for genomic selection in swine. When genotypes were imputed with lower accuracy in training and prediction animals, the accuracy of genomic evaluation was significantly decreased, and estimates of accuracy of GEBV were inflated. From our results, we can affirm that starting a genomic evaluation using low-density genotypes and a small panel of high-density haplotypes will result in reduced accuracy of evaluation. Contrarily, once an evaluation is established with a large number of animals genotyped using a high-density platform, the addition of more animals genotyped at low density is promising. Further research is needed to study the effect of adding those imputed animals to the training population in further model retraining. As mentioned previously, all code and data used in this paper has been made available through an R package, accessible at: http://tinyurl.com/MSURGEBV.

## Supplementary Material

Supporting Information
