# Supplementary material for: Accuracy of Estimation of Genomic Breeding Values in Pigs Using Low-Density Genotypes and Imputation
Source: G3 (Bethesda). 2014 Feb 13;4(4):623–31. doi: 10.1534/g3.114.010504 (PMC4059235; doi:10.1534/g3.114.010504)
Supplement: Supporting Information [file supp_4_4_623__index.html]

Accuracy of Estimation of Genomic Breeding Values in Pigs Using Low-Density Genotypes and Imputation — Supporting Information 

# Accuracy of Estimation of Genomic Breeding Values in Pigs Using Low-Density Genotypes and Imputation

## Supporting Information for Badke *et al.*, 2014

**Files in this Data Supplement:**

- Supporting Information - Figures S1-S5 (PDF, 381 KB)
- Figure S1 - Relation between the accuracy of GEBV (*rGEBV*) against the number of close and distantly related animals in the training population. (PDF, 146 KB)
- Figure S2 - Density distribution of accuracy of EBV (*rEBV*) for three traits, showing that for D250 the average *rEBV* was lower compared to the other two traits. (PDF, 137 KB)
- Figure S3 - Distribution of genomic heritability across 10 cross-validation datasets for (A) BF, (B) D250, and (C) LEA. (PDF, 136 KB)
- Figure S4 - Average accuracy of genotype imputation for imputation from a small (blue) or large (red) reference panel as a function of (A) chromosomal location of SNP and (B) MAF. (PDF, 133 KB)
- Figure S5 - Accuracy of genotype imputation in log ratio vs. the accuracy of the estimated GEBV (*rGEBV*) for (A) BF, (B) D250, and (C) LEA. (PDF, 242 KB)
