# Supplementary material for: Accuracy of Estimation of Genomic Breeding Values in Pigs Using Low-Density Genotypes and Imputation
Source: G3 (Bethesda). 2014 Feb 13;4(4):623–31. doi: 10.1534/g3.114.010504 (PMC4059235; doi:10.1534/g3.114.010504)
Supplement: Supporting Information [file supp_g3.114.010504_010504SI.pdf]

G3 · Genes | Genomes | Genetics

Accuracy of estimation of genomic breeding values in pigs using low density genotypes and imputation

Yvonne M. Badke[*], Ronald O Bates[*], Catherine W Ernst[*], Justin Fix[‡], Juan P Steibel[*,†]

[*]Department of Animal Science, Michigan State University, East Lansing, MI 48824
[†]Department of Fisheries & Wildlife, Michigan State University, East Lansing, MI 48824
[‡]Smithfield Premium Genetics Group, Rose Hill, NC 28458

Corresponding author:
Juan Pedro Steibel
Departments of Animal Science and Fisheries & Wildlife,
Michigan State University,
East Lansing, MI, USA,
Phone +1 517 432 0671
Email: steibelj@msu.edu

DOI: 10.1534/g3.114.010504

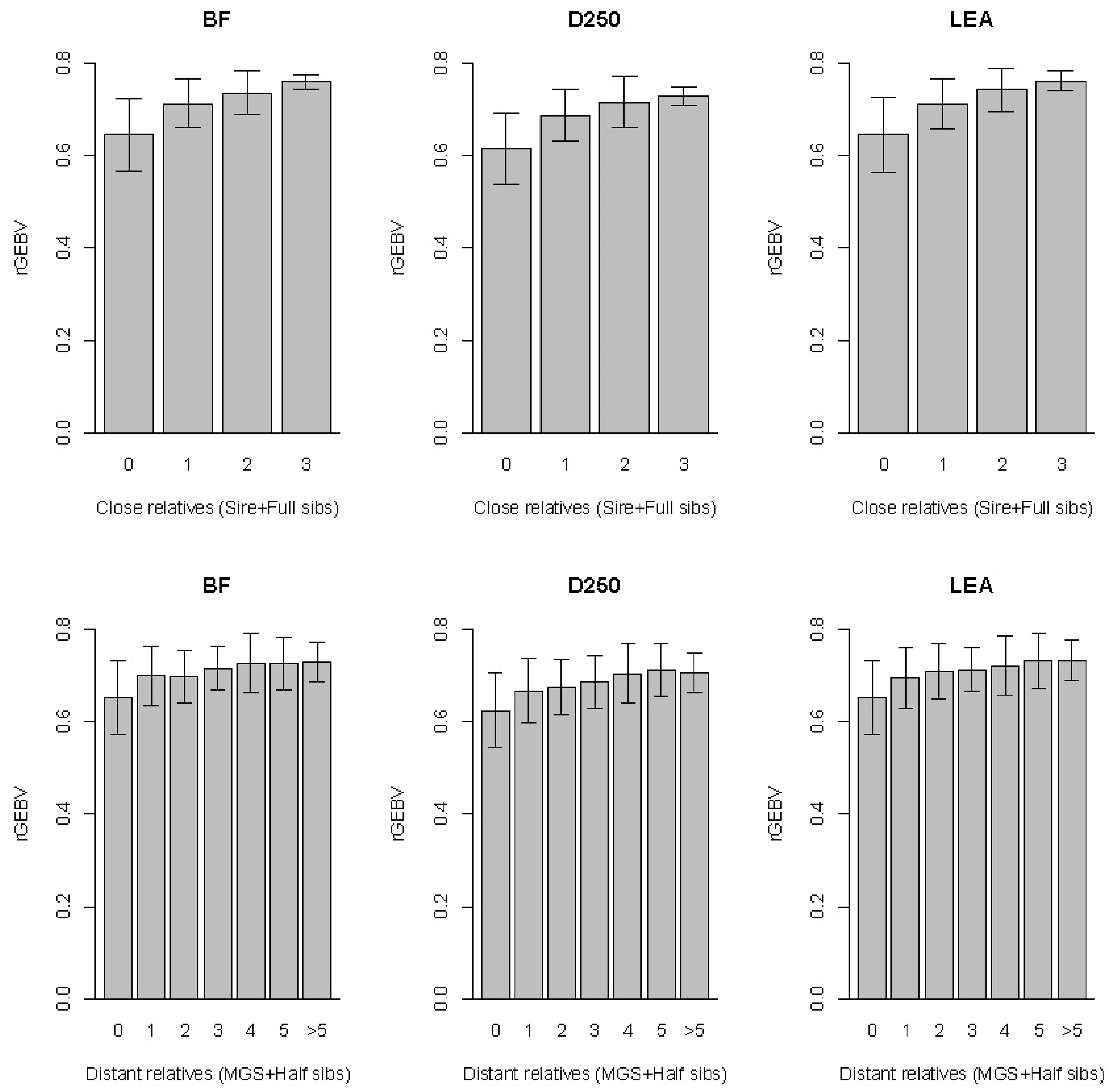

Figure S1 Relation between the accuracy of GEBV (r_{GEBV}) against the number of close and distantly related animals in the training population

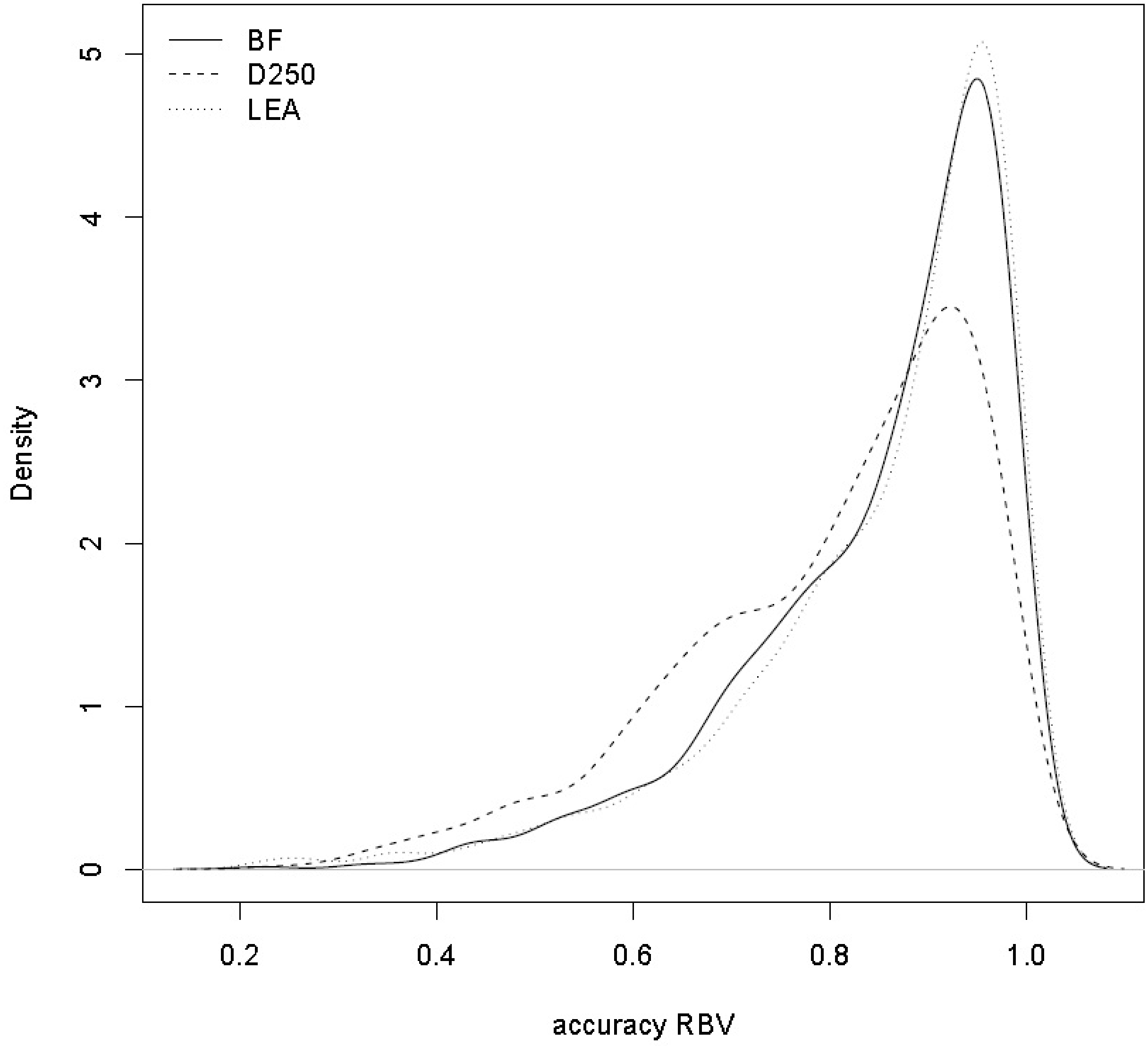

Figure S2 Density distribution of accuracy of EBV (r_{EBV}) for three traits, showing that for D250 the average r_{EBV} was lower compared to the other two traits

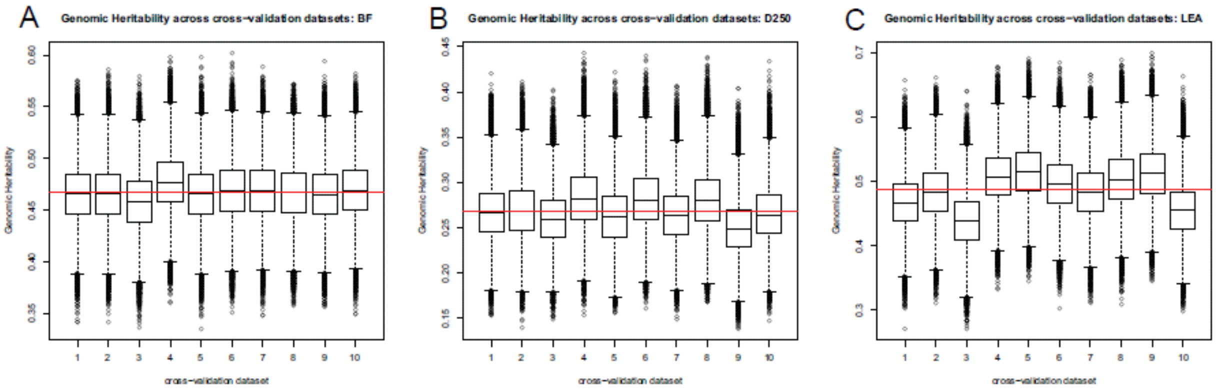

Figure S3 Distribution of genomic heritability across 10 cross-validation datasets for (A) BF, (B) D250, and (C) LEA

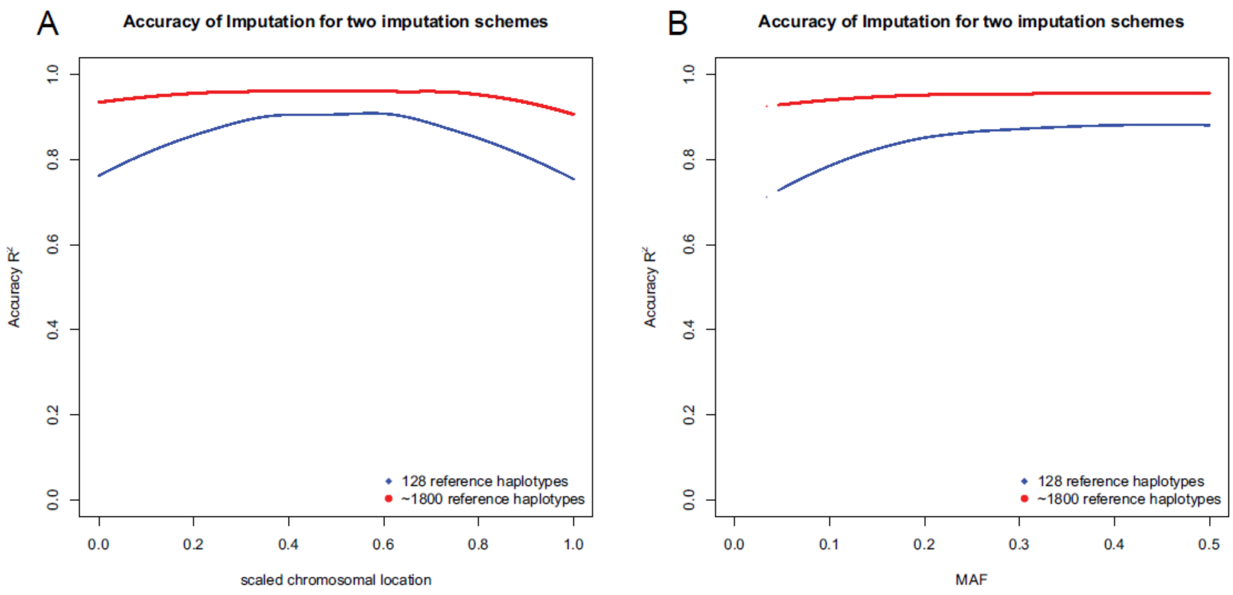

Figure S4 Average accuracy of genotype imputation for imputation from a small (blue) or large (red) reference panel as a function of (A) chromosomal location of SNP and (B) MAF

A

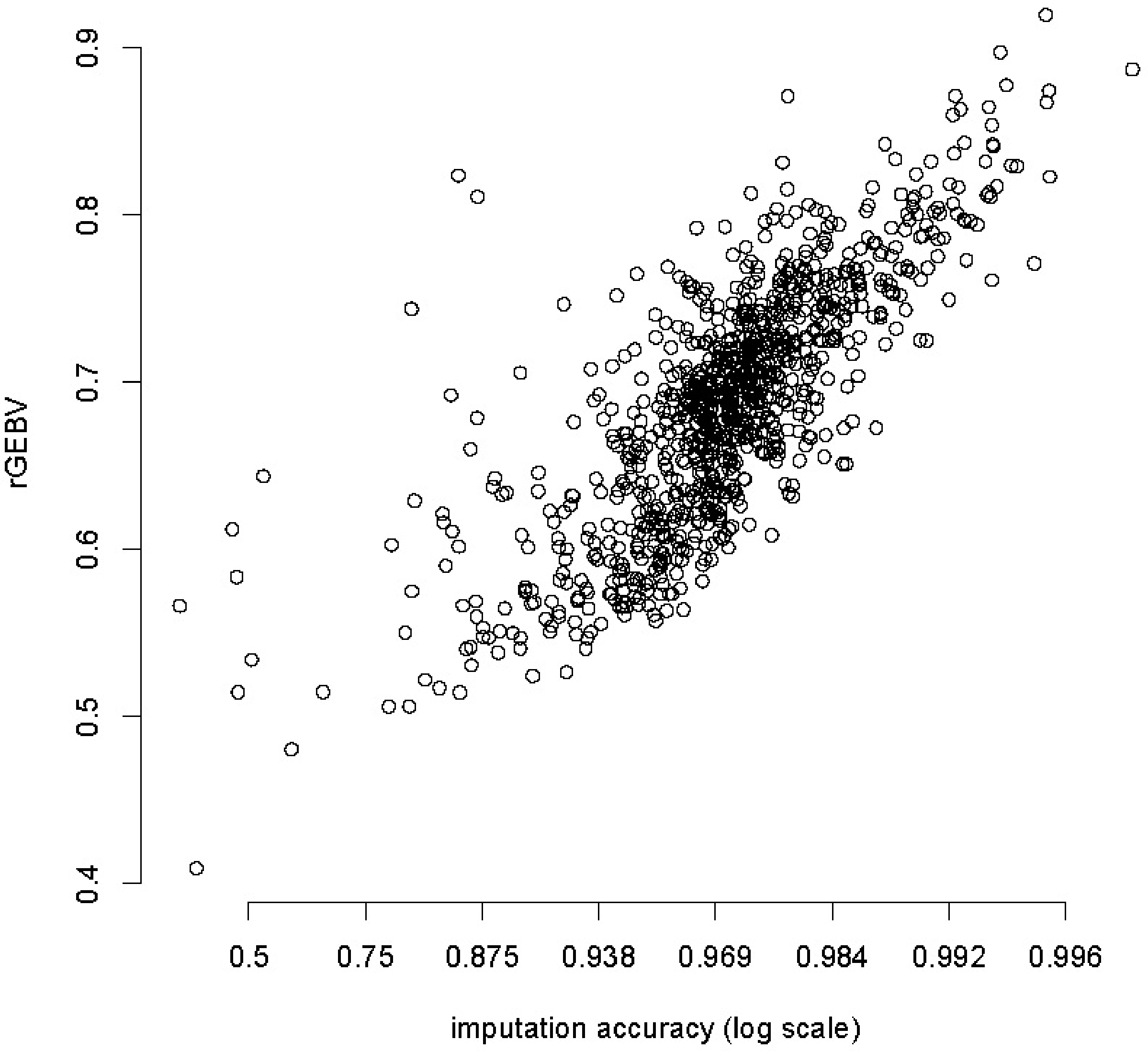
rGEBV
0.4
0.5
0.6
0.7
0.8
0.9
0.5
0.75
0.875
0.938
0.969
0.984
0.992
0.996
imputation accuracy (log scale)

B

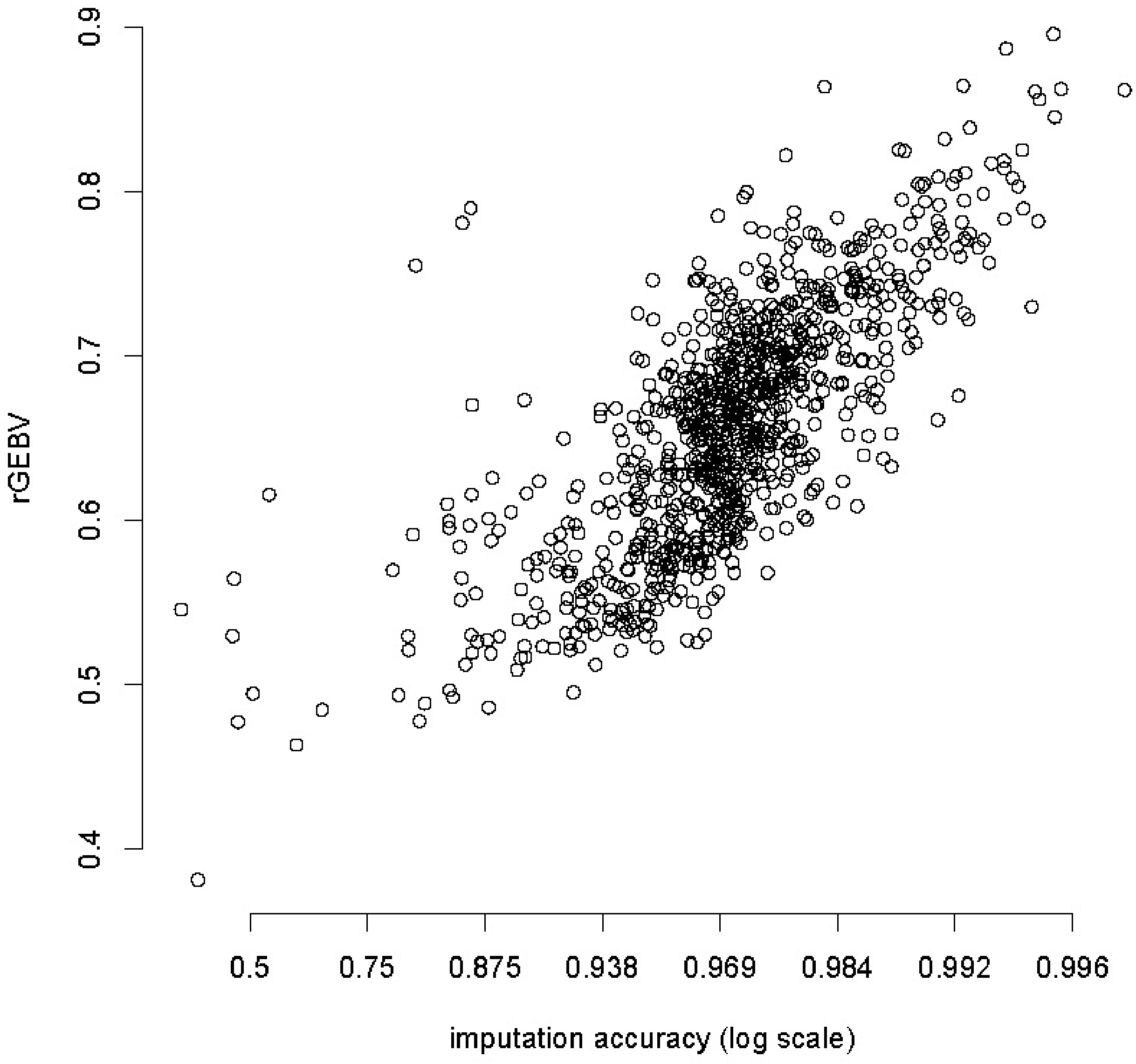

rGEBV
0.4
0.5
0.6
0.7
0.8
0.9
0.5
0.75
0.875
0.938
0.969
0.984
0.992
0.996
imputation accuracy (log scale)

C

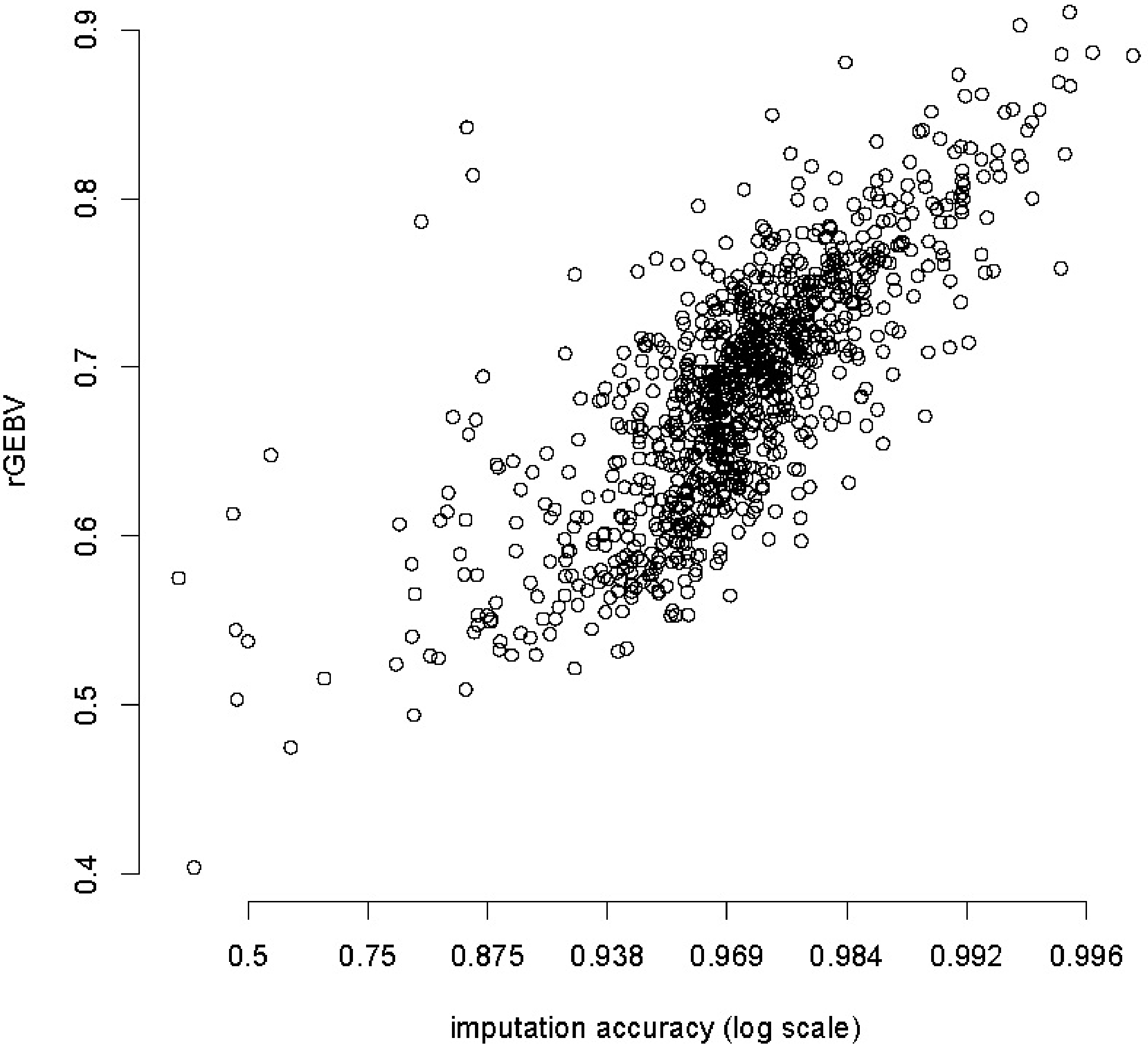

Figure S5 Accuracy of genotype imputation in log ratio vs. the accuracy of the estimated GEBV (r_GEBV) for (A) BF, (B) D250, and (C) LEA
