# Supplementary material for: Accuracy of Estimation of Genomic Breeding Values in Pigs Using Low-Density Genotypes and Imputation
Source: G3 (Bethesda). 2014 Feb 13;4(4):623–31. doi: 10.1534/g3.114.010504 (PMC4059235; doi:10.1534/g3.114.010504)
Supplement: Supporting Information [file supp_g3.114.010504_FigureS3.pdf]

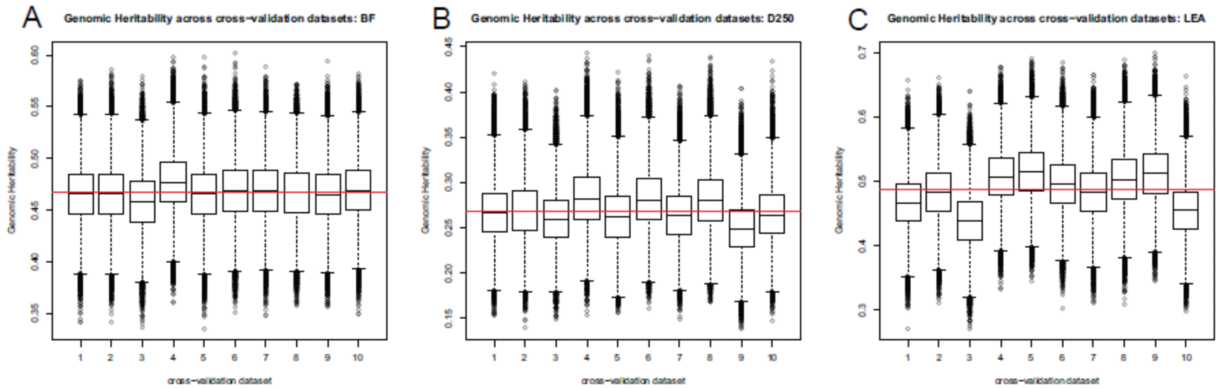

Figure S3 Distribution of genomic heritability across 10 cross-validation datasets for (A) BF, (B) D250, and (C) LEA
