# Supplementary material for: Accuracy of Estimation of Genomic Breeding Values in Pigs Using Low-Density Genotypes and Imputation
Source: G3 (Bethesda). 2014 Feb 13;4(4):623–31. doi: 10.1534/g3.114.010504 (PMC4059235; doi:10.1534/g3.114.010504)
Supplement: Supporting Information [file supp_g3.114.010504_FigureS4.pdf]

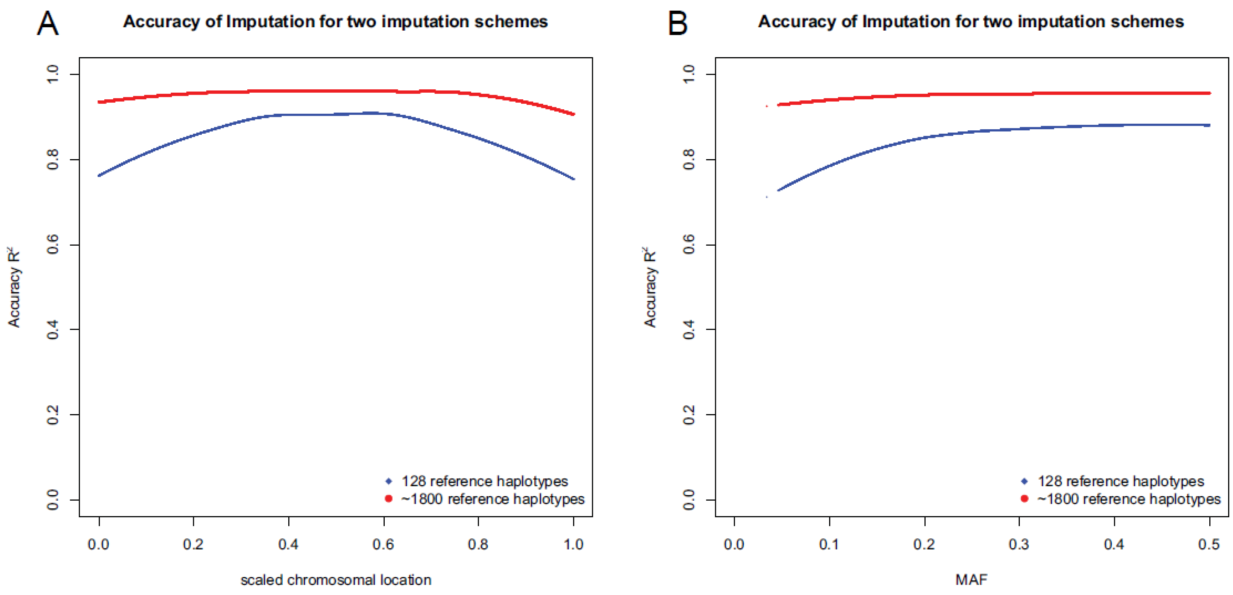

Figure S4 Average accuracy of genotype imputation for imputation from a small (blue) or large (red) reference panel as a function of (A) chromosomal location of SNP and (B) MAF
