# Supplementary material for: Accuracy of Estimation of Genomic Breeding Values in Pigs Using Low-Density Genotypes and Imputation
Source: G3 (Bethesda). 2014 Feb 13;4(4):623–31. doi: 10.1534/g3.114.010504 (PMC4059235; doi:10.1534/g3.114.010504)
Supplement: Supporting Information [file supp_g3.114.010504_FigureS5.pdf]

A

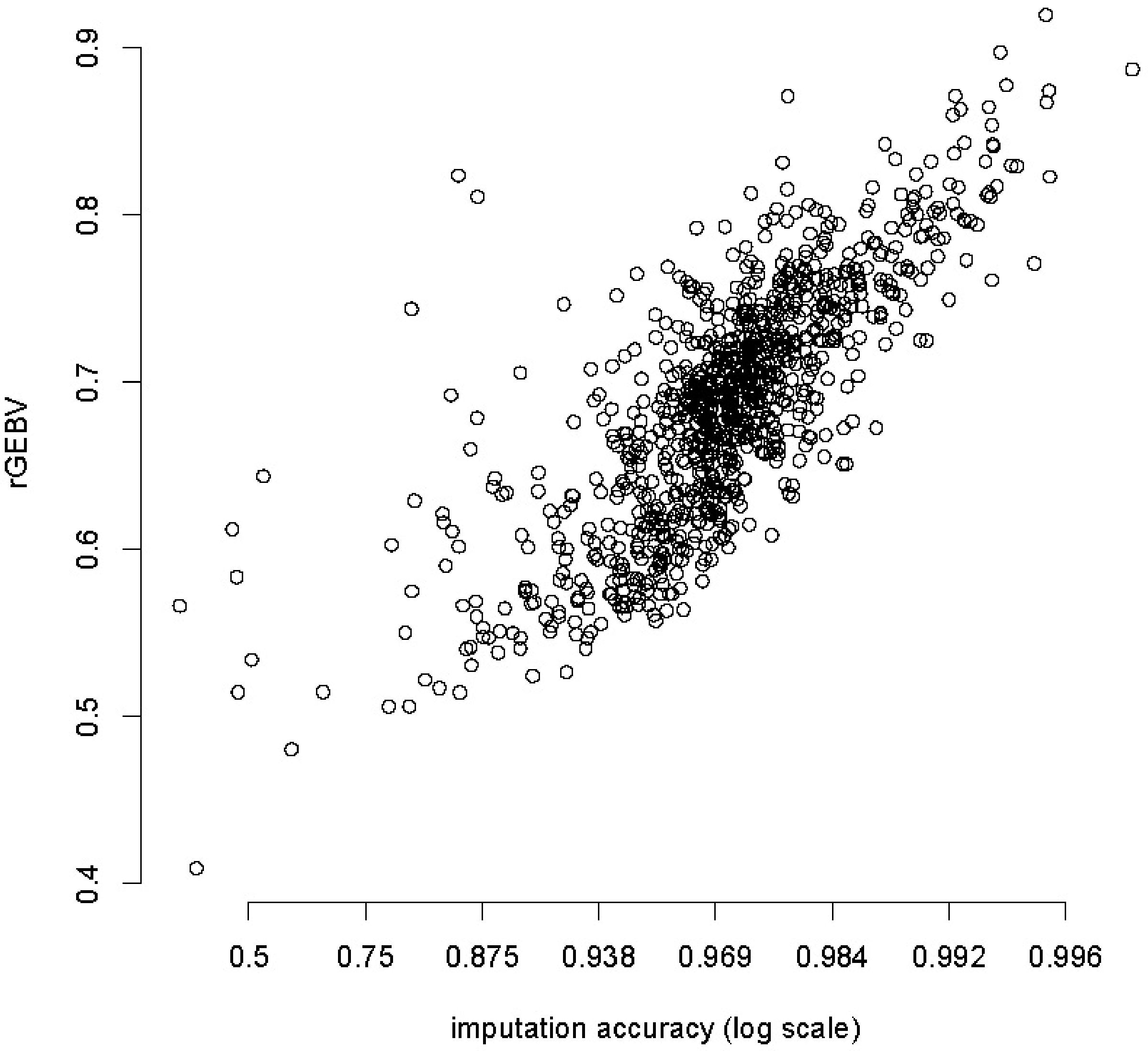
rGEBV
0.4
0.5
0.6
0.7
0.8
0.9
0.5
0.75
0.875
0.938
0.969
0.984
0.992
0.996
imputation accuracy (log scale)

B

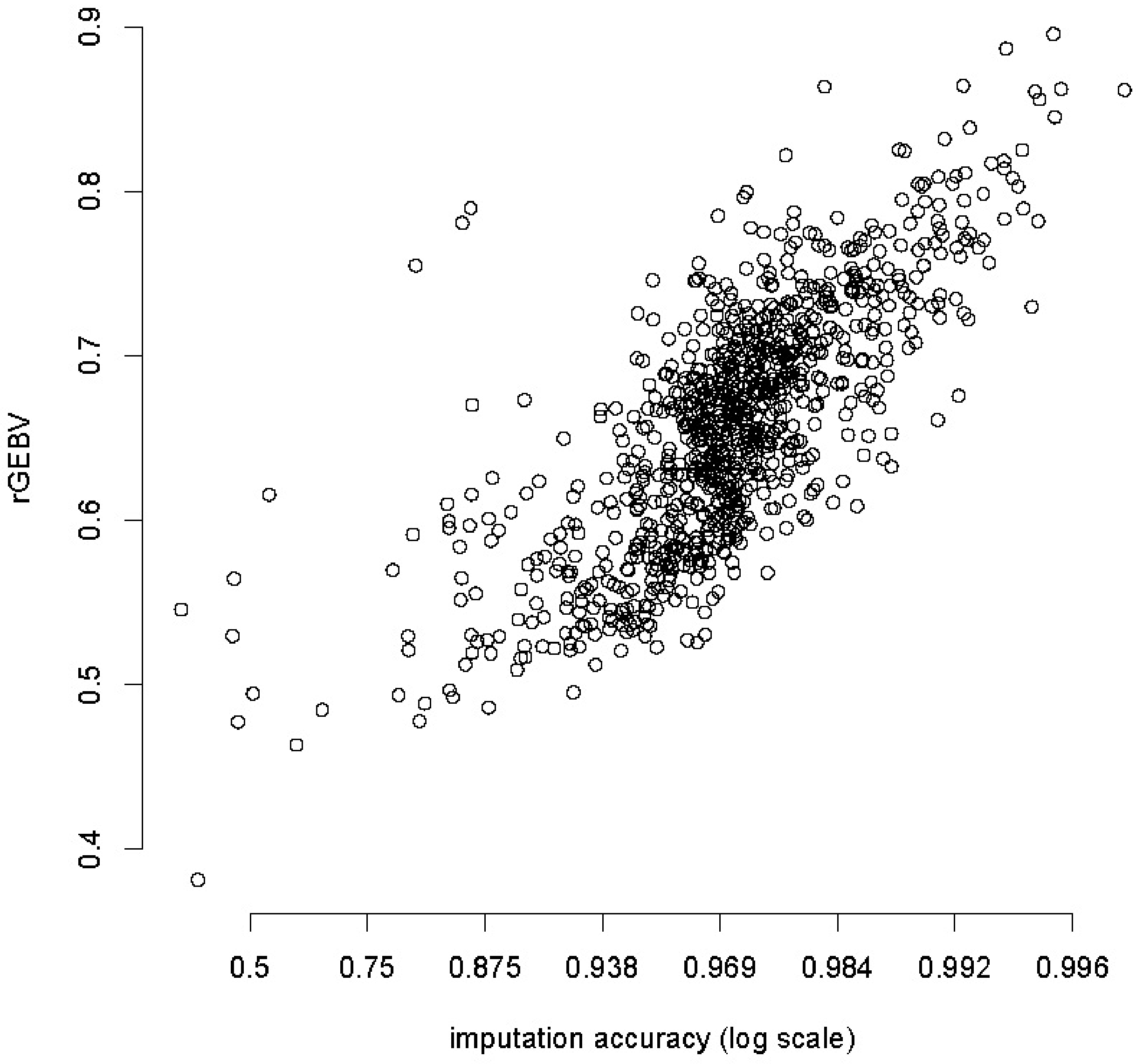

rGEBV
0.4
0.5
0.6
0.7
0.8
0.9
0.5
0.75
0.875
0.938
0.969
0.984
0.992
0.996
imputation accuracy (log scale)

C

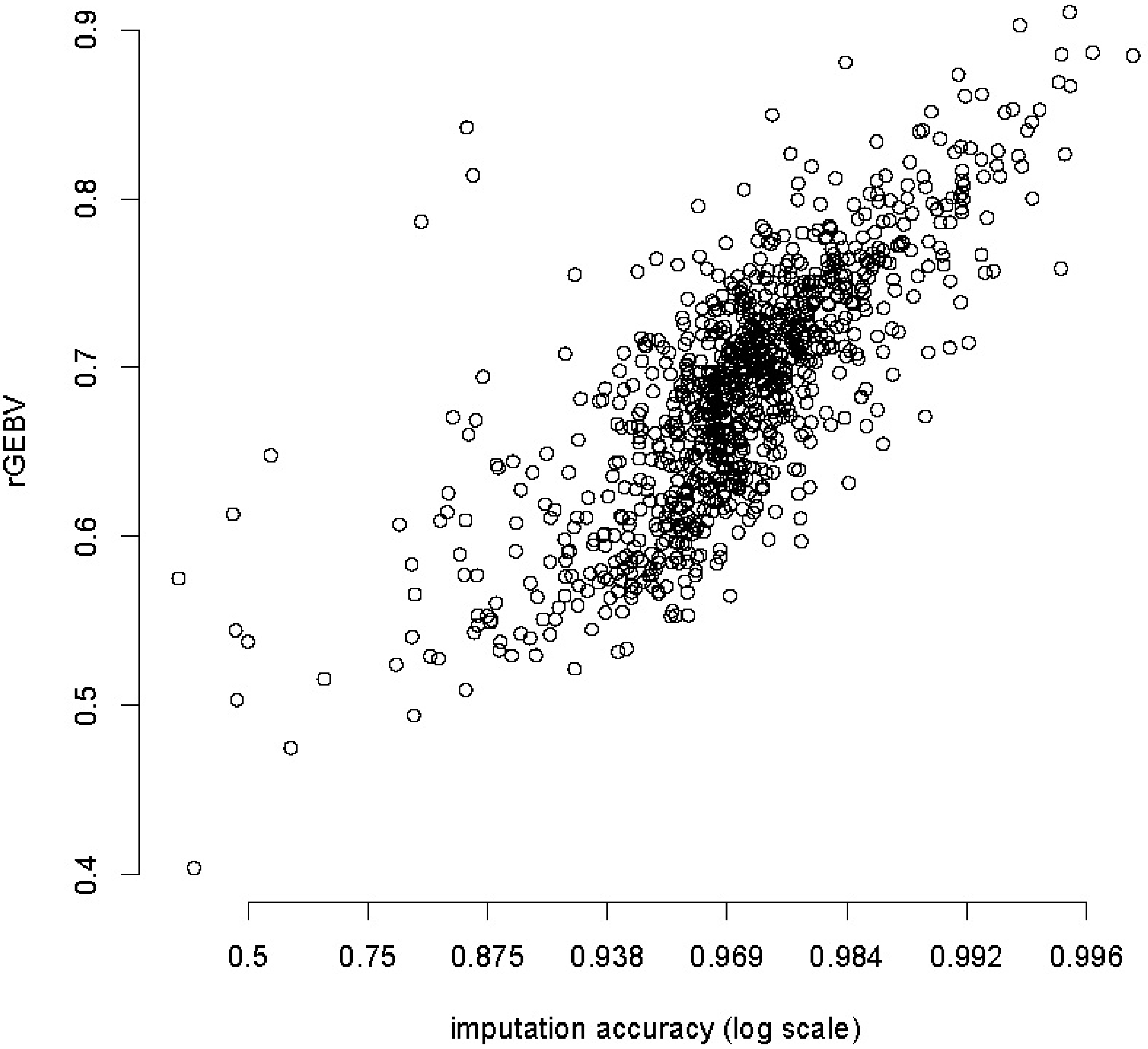

Figure S5 Accuracy of genotype imputation in log ratio vs. the accuracy of the estimated GEBV (r_GEBV) for (A) BF, (B) D250, and (C) LEA
